# Impact of Artificial Intelligence on the Care of Terminally Ill Patients

**DOI:** 10.3390/healthcare14050602

**Published:** 2026-02-27

**Authors:** Florbela Gonçalves, Margarida Gaudencio, Sofia B. Nunes, Francisca Rego, Rui Nunes

**Affiliations:** 1 Internal Medicine and Palliative Care Service, Portuguese Institute of Oncology Francisco Gentil Coimbra, 3000-075 Coimbra, Portugal; 4196@ipocoimbra.min-saude.pt; 2 Faculty of Medicine, University of Porto, 4200-219 Porto, Portugal; asnunes@med.up.pt (S.B.N.); mfrego@med.up.pt (F.R.); ruinunes@med.up.pt (R.N.)

**Keywords:** artificial intelligence, digital health, ethical dilemmas, ethics, palliative care, palliative medicine, symptom assessment

## Abstract

**Highlights:**

**What are the main findings?**

- Artificial Intelligence (AI) is a technological tool that is increasingly being used, particularly in the context of healthcare. Its use in palliative and end-of-life care presents huge potential.
- The use of AI in palliative care could help professionals with symptom control and development of communication skills, as well as assist in the construction of an advanced care plan and in shared decision-making processes.

**What are the implications of the main findings?**

- The application of AI in daily practice in palliative care is challenging, and is not without risks and ethical dilemmas.
- Even though it is becoming an increasingly valuable tool, it is important to recognize that it cannot replace human collaboration. Emotional empathy, physical comfort and compassion are qualities that AI is unable to provide.

**Abstract:**

**Introduction**: In recent decades, demographic aging has led to an inversion of the population pyramid, with a marked increase in the proportion of older adults. This shift has been accompanied by a higher prevalence of chronic and life-limiting diseases, while there have also been significant technical and scientific advances. However, these developments have not been matched by a proportional expansion of healthcare human resources, including in palliative care (PC). Consequently, healthcare systems face increasing pressure, particularly in the provision of end-of-life care. Artificial intelligence (AI) has emerged as a promising tool to support and improve healthcare delivery. **Objective**: This study aims to review the literature on the impact of AI on palliative care, with particular emphasis on its clinical applications and ethical implications in end-of-life care. **Methods**: A narrative review was conducted using a structured search of PUBMED, CINAHL and Web of Science databases, covering publications from the last ten years (2015–2025). Search terms included combinations of “artificial intelligence”, “machine learning”, “palliative care”, “end-of-life care”, and “ethics”. Articles were included if they addressed clinical applications, implementation challenges or ethical aspects of AI in PC. Reference lists of selected articles were screened to identify additional relevant studies. The findings were analyzed and synthesized thematically into key domains of application and ethical concern. **Results**: The literature suggests that AI is currently a promising tool in PC, particularly in prognostication, symptom assessment, clinical decision support, and communication. These applications may represent a paradigm shift compared to conventional approaches. However, it is important not to forget that patients in PC need much more than algorithmic decision trees. Thus, current evidence is largely exploratory, with limited real-world validation. Empathetic emotional support, physical comfort and compassion are things that artificial intelligence cannot provide. AI does not replace humans in interpersonal relationships and dignity; it only complements them. **Conclusions**: AI-based technologies hold significant potential to address contemporary challenges in PC, including inequitable access, workforce strain, and the need for more efficient service delivery. Nevertheless, their implementation raises substantial ethical concerns related to autonomy, transparency, data governance, and the preservation of human dignity. AI should therefore be understood as a complementary tool that supports—but does not replace—the human dimension of PC.

## 1. Introduction

Healthcare professionals play a fundamental role in the health system of any country. The demand for these professionals has increased significantly in recent years in many countries, including Portugal [1].

According to Health at a Glance—Europe 2024, a joint report of the Organization for Economic Co-operation and Development (OECD) and the European Commission, it was estimated that, in 2022, there was a shortage of approximately 1.2 million healthcare professionals in the countries of the European Union [2].

Artificial intelligence (AI) is a growing reality that is transforming our lives on a global scale across social, economic, educational and healthcare domains [3]. Similar changes can also be observed in scientific research and technological development [3].

The speed at which artificial intelligence has been implemented in various areas of human life is not entirely without risks [4]. Thus, while it is true that many AI tools can perform functions more effectively than humans themselves, they lack the humanistic and empathetic aspect. The implementation of AI brings discussions at the ethical and legal levels, examples of which include the lack of clarity and ambiguity of roles, the possible violation of data protection, doubts about liability for errors or biases, and concerns about free and informed consent [4,5].

Ensuring that AI is properly governed requires the establishment of a legal framework, along with continuous monitoring and regular audits, so that its ethical applicability is not called into question [5].

In the specific context of medicine, in addition to the issues above, there is an additional concern regarding the impact that AI may have on clinical practice and the delivery of care [6]. Some studies have already demonstrated that its application has limitations, particularly due to biases in the development and implementation of AI, which can distort results and reproduce or exacerbate existing social inequalities [7].

Several international organizations, such as the World Health Organization (WHO), the European Commission, the European Parliament, the Office of Science and Technology Policy (United States), and the Nuffield Council on Bioethics, have already recommended that its use be subject to oversight [7,8,9,10,11].

AI is becoming an increasingly prominent part of our lives [12]. AI involves the use of machines to perform human cognitive functions, and in many cases, these systems can surpass human capabilities [12]. AI has emerged as a highly promising tool, and its exponential growth has positively influenced organizational performance by improving efficiency and competitiveness [12].

In the healthcare field, AI is increasingly applied in diagnostic formulation, therapeutic decision-making, medical imaging analysis and the automation of administrative tasks [13]. Although AI can perform certain technical tasks at a level comparable to—or even exceeding—that of healthcare professionals, ethical considerations continue to limit its widespread implementation [13].

In the field of palliative care (PC), the progress is remarkable, creating opportunities to enhance comprehensive patient care, promote well-being and improve quality of life during advanced stages of disease [14,15].

According to the WHO, “Palliative care is an approach that improves the quality of life of patients—adults and children—and their families who are facing problems associated with life-threatening illness. It prevents and relieves suffering through the early identification, correct assessment and treatment of pain and other problems, physical, psychosocial or spiritual.” [16].

According to the World Atlas of Palliative Care (developed by WHO), around 60 million people require PC each year, yet only 12% of them have access to this type of care [17]. It is estimated that around 40 million people need PC annually worldwide due to population aging [18]. In 60% of deaths that occur globally, there is a need for PC, with deaths from cancer accounting for 34% of cases [18]. Although the relevance of early initiation of PC in the course of the disease is recognized, referral continues to be late, and in many cases death occurs without patients receiving this care. These types of situations are particularly notable in low-resource countries [19]. PC is essential to end-of-life care for cancer patients and for many individuals with non-cancer terminal illnesses [20,21,22]. Thus, in the face of the shortage of specialized human resources in PC, AI can become a valuable asset for patients in palliative care [23,24]. However, the urgent ethical dilemmas within such a noble field of medicine—and their implications at the end of life, where care should be guided by holistic and humanistic principles—raise questions about the true value of AI, despite its potential contributions pressing ethical dilemmas in such a noble area of medicine and their implications [23,24].

The aim of this article is to explore the potential applications of AI in PC, examining its benefits, implementation challenges, and key ethical considerations. In particular, it evaluates the main risks, limitations, and ethical challenges associated with integrating AI technologies into such a sensitive clinical context. This raises the following research question: “How can AI be effectively and ethically integrated into PC while mitigating its risks and limitations?”

## 2. Method

This article is a narrative literature review designed to synthesize and discuss recent studies and research concerning the application of AI in PC, particularly its impact on the care of terminally ill patients, with special attention to ethical considerations and quality of life.

The review is intended for specialists in PC and other healthcare professionals interested in the intersection of AI and end-of-life care.

A comprehensive literature search was conducted in the electronic databases PUBMED, CINAHL and Web of Science, focusing primarily on publications from the last ten years.

The search strategy included the following MESH terms and Boolean operators: (“Artificial Intelligence” OR “Digital Health”) AND (“Ethical Dilemmas” OR “Ethics”) AND (“Palliative Care” OR “Palliative Medicine”) AND “SYMPTOM ASSESSMENT”.

The search strategy was adapted according to the specific requirements of each database. To ensure comprehensive coverage of the topic, the search was complemented by relevant books, specialized publications from recognized authors, and selected authoritative websites in the field. Additionally, some essential articles were included regardless of publication date due to their foundational relevance to the subject.

After completing the database searches, all identified records were exported to Rayyan^®^ (Qatar Computing Research Institute, Doha, Qatar; https://rayyan.ai), a web-based tool designed to support the screening process in literature reviews. Duplicate records were removed within the platform. The remaining studies were screened by reviewing titles and abstracts to assess their relevance to the objectives of the review.

Articles considered potentially eligible were retrieved and analyzed in full text to ensure alignment with the proposed themes and research focus.

The inclusion criteria comprised (1) studies published primarily within the last ten years; (2) articles addressing the application of artificial intelligence in palliative care; (3) research discussing ethical aspects, quality of life, symptom management, dignity, or end-of-life care; and (4) publications written in English, Portuguese and Spanish. Foundational or seminal articles were included regardless of publication date when considered essential to the conceptual framework of the review.

Exclusion criteria included (1) studies not directly related to palliative care; (2) articles focusing exclusively on technical AI development without clinical or ethical relevance; (3) conference abstracts without full-text availability; and (4) publications in languages other than English, Portuguese and Spanish.

Data were extracted and organized according to thematic categories identified during the reading process. The findings were analyzed qualitatively, aiming to summarize, compare and critically discuss the existing evidence.

As a narrative review, this study does not follow a systematic review protocol but aims to provide a comprehensive, descriptive, and critical synthesis of the existing literature.

Since this research is based exclusively on the analysis of previously published studies and does not involve human participants, patient data, or primary data collection, ethical approval from an institutional review board or ethics committee was not required.

## 3. History and Development of Artificial Intelligence

Artificial intelligence and machine learning (ML) are emerging technologies that are transforming society and the way we perceive the world [25].

The goal of AI is to generate machines capable of mimicking the human mind [25]. AI systems have a wide range of applications, with the creation of algorithms capable of performing functions similar to those of humans at the cognitive and autonomy level [25]. ML is a subtype of artificial intelligence in which algorithms are created based on a database that enables decision-making. All these technologies allow for rapid and complex computational development, complementing human reasoning in the performance of tasks with surprising efficiency [25].

The various areas of daily application of AI and ML focus on health, education and the economy, with a significant increase in the accuracy of decisions [26]. In healthcare, for example, AI-based diagnostic and treatment tools are transforming the quality and effectiveness of patient care [26].

The availability of multidimensional data (demographic, clinical, genetic and phenotypic), combined with new technologies accessible through versatile internet-connected devices, reflects the integration of technology in healthcare and improves the delivery of care [27].

Cloud computing allows for faster and more cost-effective analysis of large amounts of data compared to traditional medical approaches [27].

Although AI is considered a recent concept, the truth is that it has existed for years. Knowing its history allows us to understand the concept and its importance today [28,29]. The idea of building machines that mimic human intelligence has existed since ancient times. However, it was only in the 20th century that the first studies on the real potential of AI began [28,29].

Alan Turing, a pioneer in computer science, published the concept of the Turing Machine, a theoretical model capable of performing any calculation by executing a program [30].

In 1943, McCulloch and Pitts presented a model of artificial neurons that attempted to mimic the biological neural network. Although the main goal was to try to understand the workings of the human mind, their work was fundamental in laying the foundations of AI [31,32]. More recently, the company OpenAI created the famous ChatGPT (OpenAI, San Francisco, CA, USA; https://chat.openai.com), a chatbot application with AI trained to hold conversations and answer questions [33].

With this historical review, it becomes clear that the idea that intelligence results from a computational process and can therefore be automated has existed for centuries [34]. There have been several objections to the notion that machines can be intelligent. In response to these criticisms, increasingly sophisticated learning techniques have been developed [34].

Despite these controversies, the beneficial and innovative role of AI in our lives is undeniable [35].

## 4. Artificial Intelligence in Healthcare

AI is a transformative field of modern computer science with the potential to reshape medical practice and healthcare [36].

Healthcare systems around the world face four major challenges: improving population healthcare, increasing patient satisfaction with health services, enhancing clinical care, and reducing health costs [37,38,39].

The application of technology in the health field is called eHealth (“electronic health”), which offers a series of tools that allow for improved performance of health systems and the quality of services provided [40]. In addition, it is considered a facilitating tool for communication, progressively expanding services in view of the rapid and continuous evolution of technology [40].

Population aging, along with the exponential growth of chronic diseases and the increase in health costs, represents a significant challenge for government systems to innovate and transform the delivery of care [41,42].

Despite more than a decade of experience using AI in healthcare, its practical applicability remains limited, with many tools still under development or discussion [43].

The exponential growth of AI applications in the 21st century, throughout society in general, has also been reflected in healthcare [44]. This phenomenon became particularly evident with the onset of the COVID-19 pandemic, during which the number of scientific publications in the main OECD countries increased from 35 in 1980 to more than 3400 in 2019 [44].

AI applications are vast, ranging from biomedical research to the management of healthcare organizations. Many hospitals already employ AI technologies to increase efficiency and performance [45,46].

These applications are being developed for virtually all areas of healthcare delivery [47]. Some algorithms are being integrated to support research, clinical decision-making, medical image analysis, and assistance in diagnostics and treatment [47].

In the field of research, its usefulness is increasingly undeniable, as AI allows for the encoding of large amounts of data and the recognition of complex patterns [48,49]. One example of AI application in this area is drug discovery. Researchers at the Massachusetts Institute of Technology (MIT) used AI techniques that enabled the discovery of molecules with antimicrobial activity. The algorithms used made it possible to identify an innovative antibiotic that allowed the cure of patients infected with multidrug-resistant microorganisms [48,49]. The advantages of AI in research are also felt at the level of clinical trials and in the selection of their participants as well as in the area of genetics, allowing for a better understanding of rare diseases and their biomarkers [48,49].

The integration of AI in the clinic is already more controversial. However, there are several medical areas in which it is beginning to prove itself a fundamental tool [48,49]. This is the case in radiology, where the algorithms developed allow for a fairly reliable interpretation of various imaging techniques, such as radiography, CT scan and MRI [50,51]. Technology develops image patterns that allow for the early detection of various diseases, such as cancer, issuing alerts and saving healthcare professionals time in viewing and interpreting images [50,51].

AI algorithms also allow for indicating the best treatment for the individual in question, taking into account their comorbidities and individual characteristics [52].

Another area of AI that has been intensively explored is robotic surgery [53,54]. With the creation of specific algorithms and the precision of the machines, it has become possible to perform surgical procedures that were previously unthinkable. This type of surgery, high-precision robotics, has become the preferred option for many surgeons given the lower risk of complications and postoperative hospitalization [53,54].

In terms of managing hospital organizations, AI has shown outstanding utility, allowing for greater accessibility and equity in patient care [55,56].

Telemedicine is promising in this context [55,56]. It allows patients who live far from healthcare facilities or who are too frail to travel to access consultations (teleconsultations) and medical prescriptions for drugs and complementary diagnostic tests (electronic prescriptions) [55,56].

However, it should be borne in mind that the recent and rapidly increasing introduction of intelligent software is not always ethically acceptable [57,58,59]. Bioethical principles such as autonomy, equity, and distributive justice can be easily questioned if their monitoring and auditing are not regulated [57,58,59].

Some population minorities such as LGBTQIA+ individuals, those facing economic hardship, the elderly, and women may be discriminated against in terms of unequal access to healthcare systems [57,58,59]. Individual variability such as gender, ethnicity, socioeconomic class, sexual orientation, and religion are characteristics that allow for a multifactorial assessment of people’s lives. Intersectionality addresses how these characteristics interact with the social, familial, and economic norms implemented in society [60]. Intersectional justice recognizes that discrimination against minorities does not act in isolation, but rather within power groups that mutually reinforce each other [61].

Cyber fraud can expose confidential patient data or circumvent informed consent. It is an ethical imperative to maintain security and trust in healthcare systems and to have a commitment to respecting patient autonomy.

In practice, AI does not replace human intelligence; instead, it augments it [62]. Human interaction in the field of medicine is irreplaceable. Therefore, AI methods should serve only as supportive tools, helping to refine clinical decision-making while maintaining a strong focus on patient-centered care [62].

## 5. Artificial Intelligence in PC and End-of-Life Care

Artificial Intelligence (AI) is increasingly becoming a part of our lives [63,64]. Although it does not have an established definition, AI can be understood as the attempt to use machines to mimic human cognitive functions that can sometimes even perform better than humans [63,64].

According to the WHO, palliative care is part of a multidisciplinary approach aimed at improving the quality of life of patients, their families, and caregivers through prevention, early identification, and reduction of suffering associated with pain or other physical, psychosocial, or spiritual conditions related to a chronic and incurable disease, which has a significant impact on the life of the patient and those around them [65,66].

In this way, palliative care addresses various pathologies, ranging from cardiovascular diseases (38.5%), cancer (34%), chronic respiratory diseases (10.3%), acquired immunodeficiency syndrome (5.7%), and diabetes (4.6%), among others [16,67]. However, access to palliative care is far from ideal, especially in low- and middle-income countries, with one possible explanation being the still common misconception that this care is reserved for those at the end of life [68]

One of the pillars of palliative care is symptom control in order to relieve the suffering of patients facing life-threatening illnesses [69]. The main goal is to improve quality of life, not to cure the disease, so effective symptom management becomes a priority [69].

One of the main reasons that lead to patients in palliative care being hospitalized is uncontrolled symptoms [69]. Hence the importance of early integration of these specialized care measures into the disease trajectory, preferably at the time of diagnosis of a life-threatening illness. This approach enables the timely identification of palliative needs that can be addressed through comprehensive early intervention. In recent decades, there has been a strong recommendation from the scientific community for palliative care to be initiated early in cancer patients, more specifically within the first 8 weeks after diagnosis [69].

It is also advocated that palliative care and curative care, such as chemotherapy, be inseparable [69]. This type of approach helps reduce symptom burden, improve quality of life, increase patient and family satisfaction, and even extend survival [69].

However, in healthcare institutions without access to PC, it is essential to identify these needs to ensure early referral [70].

There are currently several lines of research seeking to study how AI can help fill some gaps in access to palliative care [71]. AI has been seen as a valuable tool in relieving symptom burden, in decision-making, in reducing the workload of healthcare professionals, and in improving care [71].

These new technologies allow healthcare professionals to help patients in palliative care manage pain and other symptoms, potentially leading to drastic changes in the way they practice [72,73].

The potential use of AI in decision-making is also equally important in selecting the most effective and safe therapeutic options and ensuring better management of cancer patients in need of palliative care [74,75]. The integration of AI into palliative care can help improve the quality of life of terminally ill patients, as well as alleviate the suffering of the patient and family through humanistic and compassionate approaches centered on the patient [74,75].

AI is equally useful in enhancing skills in patient management in palliative care by professionals trained in the field. The practical application of insights generated by AI in end-of-life care is of utmost importance for the well-being of the patient and family [76,77].

AI allows for the identification and monitoring of symptoms. Mallick et al. designed a remote monitoring tool for symptoms in palliative care patients through the use of wearable sensors and Raspberry Pi, which allowed the measurement of vital signs such as respiratory rate and heart rate, thereby anticipating the onset of dyspnea crises in patients with advanced metastatic cancer [78].

DiMartino et al. used natural language processing (NLP) to detect uncontrolled symptoms in the digital medical records of hospitalized and end-of-life patients [79]. Shimada and Tsuneto, using RapidMiner^®^, were able to predict various symptoms such as pain, dyspnea, fatigue, anxiety, and existential distress, with accuracy ranging from 55% to 88% [80].

On the other hand, Sandham et al., through the Integrated Palliative Care Outcome Scale (IPOS) and various AI techniques (including decision trees), identified several symptoms, such as asthenia and anorexia [81].

The CLARK system used by DiMartino et al. allowed for the identification of symptomatic control issues in 61% of pain cases, 68% of nausea and vomiting cases, and 80% of dyspnea cases [82].

Kim et al. used XGBoost, Random Forest, and others as predictive models for delirium, achieving an accuracy of 69.84% [83].

AI also supports clinical decision-making [19,84]. Wilson et al. replaced common screening methods with a clinical predictive model, which was applied in a home hospitalization context, allowing earlier access to palliative care and reducing the need for patient admission [19,84].

Soltani et al. used LSTM models that allowed for the streamlining of home care services with a long waiting list [85]. Limsomwong et al., through a rule-based system with LexTo, identified patients with palliative care needs with high accuracy [86].

Terminally ill patients need a specialized palliative care team with skills in empathetic communication and compassion [87].

Van Bussel et al. studied the factors that influenced the potential acceptance of a virtual assistant and found that performance, effort, and trust were key in the acceptance decision of the server [88]. Srivastava et al. analyzed ChatGPT in palliative care communication and found it to be very similar to human communication, except regarding topics related to spirituality and death [89].

Yücel et al. compared ChatGPT with Bard and other platforms, finding that the former had a higher predictive value (96%), while Google Bard had the lowest (86%) [90].

Effective symptom management and the improvement of quality of life are the main goals of palliative care, which artificial intelligence is not unaware of [91]. AI allows pain and other symptom control to be carried out in an individualized manner in patients with advanced cancer [91].

In non-oncological contexts, Ott et al. used smart sensors for real-time monitoring of symptoms present in neurodegenerative diseases [92]. However, Deutsch et al. showed that there is a potential bias in these assessments when dealing with specific and less common non-oncological pathologies, which limits the generalization of symptom-based approaches by AI [93].

Emotional support is an essential component of palliative care, and AI could contribute through chatbots and conversational robots [94]. There are already some specific developments, such as Mabu, a robot designed to interact with patients with chronic illnesses. Mabu uses AI to maintain personalized conversations and monitor patients’ health status [94].

There has been an increase in the number of individuals with palliative care needs, which are not always adequately met due to a lack of healthcare professionals with specialized training in this area [95]. AI can assist healthcare professionals by improving prognostic accuracy, allowing for the early identification of patients at higher risk of mortality and greater symptom burden [95]. This can help avoid the use of potentially futile and disproportionate treatments in the final days of life, facilitating shared and timely decision-making with patients and their families [95].

AI uses ML techniques to assist in referring patients for PC [96,97]. ML is a subtype of AI, and its construction of predictive algorithms allows for a robust prediction of the probability of death in the context of palliative care within a time frame of between one month and five years. ML is also useful in identifying patients with palliative needs, using digital medical records as a basis [96,97].

**Figure 1:**
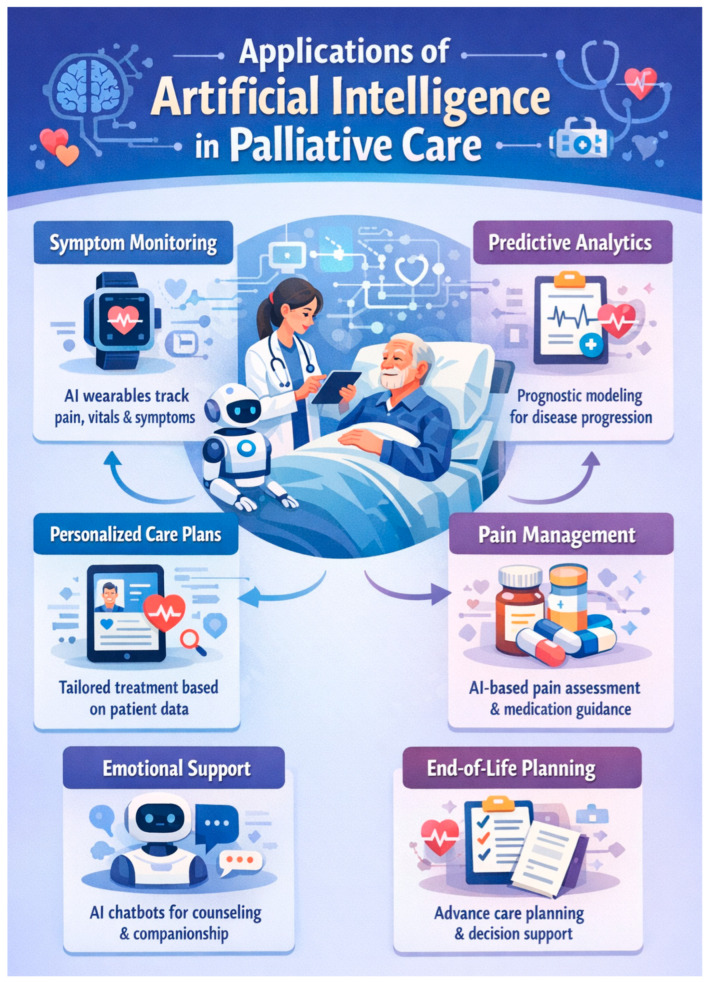
A conceptual diagram of AI’s applications in palliative care. Note: Image generated using ChatGPT^®^ on 12 January 2026.

While the usefulness of AI is undeniable (Figure 1), it cannot compromise the doctor–patient relationship or empathetic and sensitive communication when delivering bad news [98,99,100].

In palliative medicine, the goal is to provide holistic and compassionate care to patients at the end of life, ensuring their comfort and preserving their dignity [101].

## 6. Ethical Issues on the Use of AI in Palliative Care

The integration of artificial intelligence in the context of palliative care is a growing reality, allowing for the extension of life while also improving quality of life and purpose by focusing on individualized care for those in their final stages [98]. This integration must be done carefully, with zeal, to prevent the emergence of important ethical dilemmas such as the lack of privacy of individual and sensitive data, the absence of equity, and the lack of humanism in decision-making in conflicting situations [98].

With AI, traditional palliative care has transformed into a model based on evidence-based medicine [102]. Its use should always be understood as complementary and not a substitute in areas such as the anticipation of uncontrolled symptoms, the appearance of new symptoms, crises of existential suffering, individualized and patient-centered treatment, and improving care [102].

The application of predictive algorithmic models in AI allows for the early identification of palliative needs [103]. Other areas of AI application in end-of-life care include real-time symptom monitoring and the use of virtual assistants that facilitate communication between patients, family members, and healthcare professionals when face-to-face interaction is not possible [103]. These new generations of tools are very promising in improving health outcomes and access to personalized care for patients who would otherwise not have the opportunity to benefit from such a noble area of medicine as PC [103]. Despite all these virtues, we cannot forget the ethical dilemmas that can also arise when dealing with extremely vulnerable patients [103]. Thus, there are ethical imperatives that cannot be overlooked, since we are dealing with people and not merely statistical data [103]. This risk would negate the dignity and respect of patients, negatively affecting the meaning and philosophy of palliative care, which prides itself on being individualized and compassionate care offered to frail patients facing illnesses that threaten their integrity as persons [103].

The ethical issues surrounding AI in palliative care are present in several dimensions and exhibit high complexity. Ferrario et al. reinforced the idea that AI algorithmic models should be used responsibly and showed that algorithmic prediction needs to be conducted in an ethical and clean manner in healthcare in general, as well as in palliative care [104].

Ranard et al. expressed concern about the potential risk of bias in the data used by artificial intelligence algorithms, particularly in cases involving minorities, which could lead to unfounded decisions [105]. The automation of decision-making algorithms leads to biases that are not always visible when using artificial intelligence, making it difficult to explain these decisions and identify responsibilities. Algorithmic equity is essential to ensure fair and non-discriminatory decisions. The different characteristics of people can lead to different decisions, but if biases exist, the decisions will not always be the most correct.

Kay See attempted to study whether AI could serve as a consultant in bioethics, but without clear conclusions [106].

Ethicists legitimately argue that the application of AI in palliative care is consistent with the philosophy of end-of-life care and that technological advancement should not prevent the preservation of human dignity [107]. AI helps palliative care professionals make more accurate decisions in both symptom control and prognosis estimation, but its hasty integration may jeopardize patient autonomy and data privacy [107]. Without the patient’s informed consent for the use of their personal information, the use of algorithms based on this information is prohibitive [107].

The final decision always rests with the patient, family, and healthcare professionals together, and can be complemented by AI applications [23]. It is essential that healthcare professionals receive training on the ethical dilemmas of using artificial intelligence and its potential risks so that these new technologies are managed safely and effectively [23].

The application of AI in areas where human contact is essential is not at all easy. Palliative care implies communication imbued with empathy and humanism, as well as holistic patient care, so ethically it may be difficult to conceive that data privacy is not fully ensured, as is informed consent. These assumptions represent ethical imperatives in the integration of AI into palliative care.

The future of AI in PC depends on reconciling digital algorithms with complex ethical dilemmas.

It is recommended that these projects involve PC patients and their families from the outset, ensuring that their values and the cultural contexts in which they are embedded are respected and preserved.

The future use of AI in end-of-life care also depends on the critical training of professionals in digital and ethical fields, investment in research that fully combines clinical, technological, and humanistic knowledge, and the promotion of policies that ensure technological literacy and equitable access to PC. It is therefore necessary to develop responsible and impartial policies so that there is a judicious use of artificial intelligence at a global level while protecting fundamental human rights. Important policies include addressing ethical dilemmas, responsibly protecting data privacy, and encouraging public–private partnerships so that decisions are shared. To achieve this, there must be international standards that guarantee the maximization of AI benefits and the proactive regulation of its risks.

## 7. Limitations and Future Directions

According to the findings and discussions reported in the literature, AI applications in PC face several significant challenges. Data limitations are a major issue because patient records in PC are often inconsistent or limited. Privacy concerns further restrict the sharing of sensitive data.

Another key challenge is the complexity of human experience. PC addresses not only physical symptoms but also social, emotional and spiritual needs. Ethical and legal challenges are also important. End-of-life care decisions are deeply sensitive, and reliance on AI can raise questions about informed consent.

Integration and acceptance remain barriers. Healthcare professionals may hesitate to trust AI recommendations in charged situations, and incorporating AI into daily workflow can be challenging.

Despite these challenges, AI holds promise for the future of PC. Personalized symptom management could enable prediction of symptom trajectories and support tailored interventions, especially when combined with wearable devices and remote monitoring. Telemedicine and virtual care could provide 24/7 support and monitor emotional well-being, reducing the burden on healthcare providers in resource-limited settings. On the other hand, decision support tools may help clinicians in treatment planning, PC referral timing or medication adjustments while keeping final decisions human-centered. Future AI development should prioritize ethical systems, ensuring quality, equity and inclusivity.

## 8. Conclusions

Artificial intelligence has the potential to become a powerful complement to healthcare, but not a substitute for clinicians. While AI demonstrates remarkable capacity in data processing, pattern recognition, and language-based tasks, it does not possess the human attributes that are foundational to high-quality care—namely empathy, compassion, and the ability to form meaningful therapeutic relationships.

Palliative care, by definition, is holistic. It addresses the patient as a whole person across biological, psychological, social, and spiritual dimensions. This approach requires nuanced interpretation of emotional states, sensitivity to silence and nonverbal communication, and the ability to respond to suffering in ways that are deeply relational. Such human presence remains irreplaceable.

Within these boundaries, AI can play a valuable and transformative role. It can streamline administrative processes (e.g., scheduling, prescription reminders), support symptom assessment and severity classification, assist in triaging urgent cases, contribute to prognostic modeling, enhance clinical decision support, and facilitate research. In oncology-focused palliative care in particular, AI-driven prognostication and communication support tools may represent a meaningful shift from conventional approaches, offering data-informed insights that can strengthen—but not supplant—clinical reasoning.

However, important limitations and structural barriers must be acknowledged. Data fragmentation, poor interoperability between systems, incomplete integration of sociodemographic and clinical data, and challenges in secure information exchange hinder effective implementation. Moreover, as highlighted by Miralles et al. (2023), although several clinical contexts appear conducive to AI application, real-world effectiveness remains limited in many settings [102]. Technological promise does not automatically translate into practical benefit.

Ultimately, patients with advanced cancer in palliative care require far more than algorithmic decision trees. They require presence, dignity, compassion, and human connection—elements that no artificial system can authentically reproduce. The responsible integration of AI in palliative care therefore demands a balanced approach: embracing its analytical strengths while safeguarding the centrality of the human relationship. AI should enhance clinicians’ capacity to care, not distance them from the very humanity that defines palliative practice.

## Data Availability

The authors used AI tools to assist with language editing and to generate a diagram (ChatGPT^®^). The authors disclose the use of AI during the preparation of this manuscript, and they are fully responsible for the accuracy, integrity and originality of the data review.

## Abbreviations

AI—Artificial Intelligence; CT—Computed Tomography; IPOS—Integrated Palliative Care Outcome Scale; LGBTQIA+—Lesbian, Gay, Bisexual, Transgender, Queer, Intersex, Asexual, plus; LSTM—Long Short-Term Memory; MIT—Massachusetts Institute of Technology; ML—Machine Learning; MRI—Magnetic Resonance Imaging; NLP—Natural Language Processing; OECD—Organization for Economic Co-operation and Development; PC—Palliative Care; WHO—World Health Organization.
